# Design of Experiments for Optimizing Ultrasound-Assisted Extraction of Bioactive Compounds from Plant-Based Sources

**DOI:** 10.3390/molecules28237752

**Published:** 2023-11-24

**Authors:** Luis Miguel Anaya-Esparza, Edward F. Aurora-Vigo, Zuamí Villagrán, Ernesto Rodríguez-Lafitte, José Martín Ruvalcaba-Gómez, Miguel Ángel Solano-Cornejo, Victor Manuel Zamora-Gasga, Efigenia Montalvo-González, Horacio Gómez-Rodríguez, César Eduardo Aceves-Aldrete, Napoleón González-Silva

**Affiliations:** 1 Centro Universitario de los Altos, Universidad de Guadalajara, Tepatitlán de Morelos 47620, Mexico; blanca.villagran@academicos.udg.mx (Z.V.); horacio.gomez@cualtos.udg.mx (H.G.-R.); caceves@cualtos.udg.mx (C.E.A.-A.); napoleon.gonzalez@cualtos.udg.mx (N.G.-S.); 2 Escuela de Ingeniería Agroindustrial y Comercio Exterior, Universidad Señor de Sipán, Chiclayo 14000, Peru; rlafitte@uss.edu.pe (E.R.-L.); masolano@uss.edu.pe (M.Á.S.-C.); 3 Centro Nacional de Recursos Genéticos, Instituto Nacional de Investigaciones Forestales, Agrícolas y Pecuarias, Tepatitlán de Morelos 47600, Mexico; ruvalcaba.josemartin@inifap.gob.mx; 4 Laboratorio Integral de Investigación en Alimentos, Tecnológico Nacional de México/Instituto Tecnológico de Tepic, Tepic 63175, Mexico; vzamora@ittepic.edu.mx (V.M.Z.-G.); emontalvo@ittepic.edu.mx (E.M.-G.)

**Keywords:** ultrasound, phytochemicals, phenolic compounds, green extraction, DOE, mathematical models, desirability function

## Abstract

Plant-based materials are an important source of bioactive compounds (BC) with interesting industrial applications. Therefore, adequate experimental strategies for maximizing their recovery yield are required. Among all procedures for extracting BC (maceration, Soxhlet, hydro-distillation, pulsed-electric field, enzyme, microwave, high hydrostatic pressure, and supercritical fluids), the ultrasound-assisted extraction (UAE) highlighted as an advanced, cost-efficient, eco-friendly, and sustainable alternative for recovering BC (polyphenols, flavonoids, anthocyanins, and carotenoids) from plant sources with higher yields. However, the UAE efficiency is influenced by several factors, including operational variables and extraction process (frequency, amplitude, ultrasonic power, pulse cycle, type of solvent, extraction time, solvent-to-solid ratio, pH, particle size, and temperature) that exert an impact on the molecular structures of targeted molecules, leading to variations in their biological properties. In this context, a diverse design of experiments (DOEs), including full or fractional factorial, Plackett–Burman, Box-Behnken, Central composite, Taguchi, Mixture, D-optimal, and Doehlert have been investigated alone and in combination to optimize the UAE of BC from plant-based materials, using the response surface methodology and mathematical models in a simple or multi-factorial/multi-response approach. The present review summarizes the advantages and limitations of the most common DOEs investigated to optimize the UAE of bioactive compounds from plant-based materials.

## 1. Introduction

Food losses and food waste are growing concern that significantly affects the environment and the sustainability of the food agroindustry. In developed nations, food loss occurs when supply exceeds demand. However, in less developed countries, the lack of infrastructure or adequate preservation and handling methods causes food losses to increase significantly each year, ranging from 30 to 60%. This fact, in turn, leads to environmental problems due to the generation of food waste and economic issues resulting from the waste of resources that could otherwise be used to improve food production and food security [1]. In this context, plant-based agro-industrial foods, such as fruits, vegetables, tubers, roots, leaves, edible flowers, and their by-products (peels, seeds, and columella), have the highest incidence of wastage. Globally, 40 to 50% of all food waste comes from these natural sources [2,3]. On the other hand, plant-based sources contain secondary metabolites and biologically active phytochemicals with high added value, which can be utilized in cosmetics, nutraceuticals, pharmaceuticals, and food industrial applications [4]. This strategy not only contributes to reducing food waste, one of the Sustainable Development Goals of the United Nations 2030 Agenda [5], but also aligns with the principles of the circular economy (recycle, recovery, and reutilize) [6].

Secondary metabolites are compounds synthesized by plants through different metabolic pathways, such as shikimic acid, malonic acid, mevalonic acid, and non-mevalonate pathways. These compounds serve as signaling molecules or defense agents against predators, pests, and microorganisms, but they are not essential for the growth and development of plants [7,8]. The most representative secondary plant metabolites include phenolic acids, flavonoids, carotenoids, alkaloids, stilbenes, saponins, and terpenes [9]. From another perspective, these phytochemicals are also known as bioactive compounds, defined as “inherent non-nutrient constituents of food plants with anticipated health-promoting/beneficial and/or toxic effects when ingested” [10]. In this context, bioactive compounds have potential technological uses associated with their biological activities, such as antioxidant, antimicrobial, antidiabetic, anti-inflammatory, and antitumoral effects, among others [11].

Bioactive compounds (BC) can be extracted from plant-based materials using various extraction methods [3,12]. Traditionally, phytochemicals have been extracted through conventional solid-liquid extraction techniques, such as maceration, Soxhlet extraction, and hydro-distillation [8,13]. However, these methods are time-consuming, require large quantities of expensive and high-purity solvents, and often yield low extraction selectivity and efficiency [14]. In light of these drawbacks associated with conventional extraction methods, non-conventional or emerging extraction technologies, including pulsed-electric field-assisted, enzyme-assisted, microwave-assisted, high hydrostatic pressure, pressurized and supercritical fluids, and ultrasound-assisted methods, have been developed [8,13,15]. Among these, ultrasound-assisted extraction (UAE) is one of the most utilized techniques for extracting BC from plant-based agro-industrial foods [16,17,18].

The application of UAE has emerged as an advanced, cost-efficient, eco-friendly, and sustainable alternative to conventional extraction methods for obtaining BC from plant sources with higher yields [14,18,19]. UAE has been recently employed for extracting BC from a variety of natural sources, including pumpkin peel [1], edible mushrooms [16,17], garlic leaves [20], and raspberries [21]. Ultrasound waves have been found to facilitate the release of BC from the plant-based matrix to the medium by causing physical and mechanical changes in cell walls through a phenomenon known as acoustic cavitation [1]. It leads to an increase in extraction yield while simultaneously reducing extraction time, energy consumption, and solvent usage [20]. However, the efficiency of UAE is influenced by numerous factors within the extraction process, including the type of solvent, extraction time, solvent-to-solid ratio, pH, and temperature. Additionally, operating variables such as frequency, amplitude, and ultrasonic power play a critical role [21,22]. These variables can also impact the molecular structures of the targeted compounds, resulting in variations in their biological properties. Consequently, it is essential to define the optimal UAE experimental conditions for each specific plant-based material to maximize the recovery of BC [16]. To address this need, a range of statistical experimental designs, including Full factorial, Fractional factorial, Plackett–Burman, Box-Behnken, Central composite, Taguchi, Mixture, D-optimal, and Doehlert designs, have been investigated in UAE processes. These designs, along with predictive statistical tools based on mathematical models such as response surface methodology, aim to optimize extraction conditions and generalize experimental results. This approach makes it feasible to understand the potential for process scale-up [16,19,21,23,24,25].

The present review summarizes the advantages and limitations of the most common Design of Experiments investigated to optimize the UAE of BC from plant-based materials.

## 2. Extraction Methods of Bioactive Compounds from Plant Sources

The solid-liquid extraction of BC from plant materials is often considered the initial and crucial unit operation when developing technological products or ingredients for various industrial purposes [26]. There are different extraction approaches available for extracting BC from plant-based sources, which include both conventional and non-conventional methods. Conventional extraction methods, such as maceration, Soxhlet, and hydro-distillation, are simple and easy to execute, relying on solid-liquid interactions. However, these methods have some significant limitations, such as lower efficiency due to their extended extraction times, lower yields, and the extensive use of costly solvents [16]. Additionally, these techniques can potentially lead to the degradation of BC through processes such as oxidation, ionization, and hydrolysis [27].

In order to solve the limitations of conventional extraction methods, non-conventional-assisted extraction techniques have been under investigation for the past 50 years [8]. These extraction methods are often called green extraction technologies, which adhere to the six principles of “Green Extraction”. These principles include the following: (i) innovation through the selection of plant varieties and the use of renewable resources; (ii) utilization of alternative solvents, primarily water or agro-solvents; (iii) reduction in energy consumption through energy recovery and the adoption of innovative technologies; (iv) promotion of co-products instead of waste, thus involving the bio- and agro-refining industry; (v) minimization of the number of unit operations and the development of safe, robust, and controlled processes; and (vi) aiming for non-denatured and biodegradable extracts free from contaminants [28,29]. In this context, modern-assisted extraction methods have demonstrated significant advantages, including reduced extraction times, low energy and solvent consumption, high recovery yields of BC, and a reduced environmental impact. These techniques employ various approaches, including enzymes, pulsed-electric fields, microwaves, supercritical and pressurized fluids, and ultrasound [30]. A comparative example of the recovery yield of total phenolic compounds from *Psidium cattleianum* leaves using different conventional and non-conventional extraction technologies is presented in Table 1, where non-conventional-assisted extraction techniques consistently show higher yields compared to conventional methods, with UAE particularly standing out [31].

## 3. Ultrasound-Assisted Extraction

UAE is a green, efficient, low-cost, affordable, and user-friendly method for extracting BC from plant-based sources [18,21]. It is a technique that relies on applying ultrasound in a medium, primarily liquid, enabling the creation of cavitation bubbles through the compression and expansion of ultrasound waves. These bubbles eventually collapse, generating shock waves [16,20]. Acoustic cavitation is the primary physical phenomenon associated with ultrasound [4]. It can manifest as either transient (resulting in mechanical effects, such as high pressures and temperatures) or stable (causing shear stress due to micro-jets resulting from the implosion of microbubbles surrounding the cells) [38,39]. This phenomenon plays a crucial role in breaking down cell walls, enhancing the diffusion of solvents into plant tissues and increasing mass transfer, ultimately leading to improved release and higher yields of extractable BC [40].

UAE can be applied directly using an ultrasonic probe or indirectly through an ultrasonic bath, depending on the configuration of the ultrasound devices. However, direct ultrasound application is generally more potent and efficient due to its concentrated effect in a specific area [41]. On the other hand, in ultrasonic baths, the strength should be high enough to induce cavitation within the bath extraction vessel; nonetheless, the extraction vessel should be placed just above the transducer to guarantee that the ultrasonic power is well distributed within the system [18]. Additionally, the UAE method is versatile and can be employed with polar and non-polar solvents at different temperatures to enhance the yield of phytochemicals while reducing extraction time [4,16]. Furthermore, the ultrasound can be simultaneously applied with other extraction methods to improve the recovery efficiency of BC from plant-based materials, including heat [42], vacuum pressure [43], high pressure [44], enzyme [45], pulsed electric field [46], pressurized liquids [47], and microwave [48] among others. UAE has found successful application in a wide range of plant-based sources for recovering BC, including by-products from oranges, limes, and tangerines [49,50], *Ficaria kochii* flowers [51], *Morus alba* leaves [52], peaches and pumpkins [53], green tea leaves [54], *Hibiscus sabdariffa* calyces [55], and banana peel [56], among others [57,58,59].

Several studies have highlighted the need for different UAE conditions for various plant-based matrices to maximize the extraction yield of the target compound [20,21,31,40]. Variables such as power intensity, frequency, and ultrasound amplitude during the UAE process [60,61,62], as well as the type of solvent, extraction time, solvent-to-solid ratio, pH, and temperature [63,64], have been reported to influence extraction efficiency. Given the absence of universally defined ideal conditions for extracting BC from plant-based sources, optimizing the extraction conditions for each specific plant material becomes imperative to enhance the efficiency of the UAE process [4] and achieve the highest possible yield, both in terms of quality and quantity of BC from any food matrix. This optimization not only improves results but also minimizes workload, resource consumption, and energy use [65]. In this context, various statistical experimental designs and tools based on mathematical models have been explored to predict outcomes, facilitating the generalization of acquired experimental data and providing insights into the potential for scaling up the UAE process [2,17,20,21].

## 4. Common Statistical Tools Used for Optimizing and Modeling UAE of Bioactive Compounds form Plat-Based Materials

The design of experiments (DOEs) is a statistical methodology applied to support the design, development, and optimization of processes [66]. DOEs encompasses a collection of multivariate mathematical and statistical techniques to examine a system’s behavior by manipulating the levels of variables that influence it [67]. Statistical experimental designs provide essential insights, converting inputs into outputs and facilitating the optimization of complex processes, including those related to various plant-based materials using UAE [16]. Statistical designs can be categorized based on their objectives and scope. The selection of an appropriate design is influenced by factors such as the experiment’s purpose, the number of factors and levels to be studied, the effects under investigation, and the cost of conducting the experiments [26,58]. Depending on their objectives, statistical designs can compare two or more treatments, assess the impact of different factors on the response, and optimize various processes [20]. Furthermore, the optimization methods can be performed in two stages: (i) a screening step in which a large number of factors are analyzed to find those that have a substantial impact on essential variables, and (ii) an optimization step in which additional factor analysis is performed to identify the optimal analytical conditions [68].

In this context, these statistical tools are based on modeling complex relationships between factors and quantitative response variables in a univariate or multivariate approach to predict outcomes based on these relationships [20]. The most common statistical experimental designs investigated for optimizing UAE processes include full and fractionated factorial designs [17,50], Box-Behnken designs [52,59], central composite designs [51,69], Taguchi designs [24,70], Plackett–Burman designs [71,72], and combined designs for screening [73]. Most of these statistical designs are optimized using response surface methodology and polynomial equations [27,31,74,75], as discussed below.

### 4.1. Response Surface Methodology

Modeling has become a prevalent and effective tool in recent years for reducing the costs and time associated with conducting extensive experiments. Mathematical modeling enables the simulation, optimization, design, and control of processes [76]. The application of the DOE and Response Surface Methodology (RSM) encompasses various stages, including experiment planning, execution, result analysis, and validation of predictions against experimental values [67]. RSM plays a significant role in optimizing complex processes such as UAE for recovering biomolecules from plant-based sources [77,78]. RSM is a set of mathematical and statistical tools commonly used to investigate the impact of UAE parameters, model them, and optimize their settings [79]. This methodology allows for the calculation and assessment of experimental errors [77,80]. RSM can assess multiple parameters and their interactions on the response using mathematical models, typically represented by second-order polynomial equations (Equation (1)), requiring only a small number of experiments [40,52]. This capability to determine the optimal UAE conditions for achieving maximum yields of BC from natural sources is a key advantage of RSM over classical approaches [4,81].

(1) $$Y=b_{0}+b_{1}X_{1}+b_{2}X_{2}+b_{3}X_{3}+b_{11}X_{1}^{2}+b_{22}X_{2}^{2}+b_{33}X_{3}^{2}+b_{12}X_{1}X_{2}+b_{13}X_{1}X_{3}+b_{23}X_{2}X_{3}$$

*Y* = predicted response; *X* = independent variable; *b*_1_, *b*_2_, and *b*_3_ = regression coefficients for the linear effect term; *b*_11_, *b*_22_, and *b*_33_ = quadratic effect terms; *b*_12_, *b*_13_, and *b*_23_ = linear interaction effect terms [82].

Optimizing UAE using an RSM approach generally involves six key steps [83]:

1. Select the independent variables and their respective levels, along with potential response variables. At this stage, a screening design of experiments (DOE) can be employed.
2. Choose the appropriate DOE.
3. Conduct experiments and record the results.
4. Develop a model equation based on the experimental data, which can be visualized as a contour plot or a 3D surface and Paret chart.
5. Validate the model. This step often employs analysis of variance to identify the most significant factors in the model and assess their reliability.
6. Determine the optimal conditions.

RSM, in general, is a predictive tool that analyzes the interactions between factors and responses, enabling process optimization with a reduced number of experiments [84]. In this context, RSM has been utilized to enhance the UAE process for extracting phenolic compounds from various agro-industry by-products. These include black locust flowers [84], red cabbage [70], orange peel [85], beech bark [86], walnut male flowers [87], and *Nephelium lappaceum* husk [88], among others. Different experimental designs have been applied, such as full factorial design [89], fractional factorial design [90], Plackett Burman design [91], Box-Behnken design [92], central composite design [2], and others, as discussed below.

The DOE is commonly used for optimizing processes, including UAE. However, when the optimization process involves multiple responses (more than one), it becomes impractical to optimize each response individually since the number of solutions equals the number of variables under investigation [68]. In such scenarios, one solution for optimizing simultaneously multiple responses is to employ the Desirability function (D), developed by Derringer and Suich in 1980 [68,93]. This function is designed to identify operating conditions that not only meet the requirements of all the involved responses but also provide the best compromise values for the overall desired response. It achieves this by transforming multiple response problems into a single response, with desirability scores ranging from 0 (indicating an undesirable response) to 1 (representing a highly desirable value) [94]. The multivariate response approach takes less time, effort, and resources than the univariate procedures.

### 4.2. Full Factorial Design

Full factorial designs (FFD) involve the examination of all possible combinations of factors and levels that can be explored. This statistical tool provides a comprehensive understanding of process behavior. In FFD, all factors and their interactions are considered. There are various types of FFD, including those with two, three, and four levels, each offering different insights [66]. The two-level FFD (2*^k^*, where *k* represents the number of factors) is the most used, followed by the three-level FFD. This approach is known for its robustness and adaptability and is particularly useful when there is suspicion of interactions between factors (*X_i_* and *X_j_*) [95]. However, for the application of this method, the number of factors should fall within the range of 2 to 5 (2 < *k* < 5) to generate between 4 to 32 experimental runs. For instance, a full two-level factorial design for four factors would require 2^4^ = 16 experiments. In general, the significance of the factors and their interactions is analyzed under the following model (Equation (2)):

(2) $$Y_{ijk}=µ+\alpha_{i}+\beta_{j}+{\left(\alpha \beta \right)}_{ij}+\varepsilon_{ijk}$$

where µ is the overall average, $\alpha_{i}$ is the effect due to the *i*-th level of factor A, $\beta_{j}$ is the effect due to the *i*-th level of factor *β*, ${\left(\alpha \beta \right)}_{ij}$ represents the interaction effect in the combination *ij*, $\varepsilon_{ijk}$ is the random error that is assumed to follow a normal distribution with a mean of zero and constant variance [66].

The FFD have been employed to assess the impact of several factors and their respective levels in UAE processes aimed at recovering BC from plant-based sources (Table 2).

In factorial designs, factors and their respective levels are intentionally and simultaneously manipulated to assess their main effects on the variable response [100]. In the context of UAE for extracting polyphenols from beech bark (using a full factorial design of 3^3^, RSM, and a quadratic model with R^2^ = 0.78), the interactions between time-solvent and temperature-solvent significantly influenced the extraction yield. Surprisingly, the interaction between temperature and time had no effect. The optimal UAE conditions for achieving the highest polyphenol extraction (67.87 mg/g) were 70% ethanol at 65 °C for 20 min [86]. Similar trends were observed during the UAE of polyphenols from spruce wood bark (using a full factorial design of 3^3^ with 2 replications, RSM, and a quadratic model with R^2^ = 0.92). The optimal extraction conditions for maximizing the yield (13.23 mg/g) were 70% ethanol and a temperature of 54 °C for 60 min. However, the factors’ effects could be ranked as follows: ethanol concentration > extraction time > temperature [89]. It has been noted that during UAE, the combined effects of temperature and solvent concentration enhance solvent mass transfer, facilitating the release of polyphenolic compounds from the food matrix [86,96]. These effects are associated with changes in viscosity, solubility, polarity, and surface tension within the solvent and food matrix [80,88]. However, prolonged extraction times can lead to polyphenol degradation, emphasizing the importance of determining an optimal extraction duration [22]. On the other hand, it has been reported that the extraction time had no significant effect, even when using a 60 kHz frequency for 30 min, on the recovery of phenolic compounds from lime, orange, and tangerine peels under UAE (ultrasonic bath) [50].

Furthermore, in the context of UAE for extracting polyphenols from rambutan husk (using a full factorial design of 3^3^), it was observed that, in addition to extraction time and ethanol concentration, the extraction yield was significantly affected by the solid-to-liquid ratio, which is associated with the interaction between solute and solvent. The highest yield of phenolic compounds (487.67 mg/g) exhibiting strong antioxidant capacity (ABTS 92.50% and DPPH 73.73%) was achieved when using a mass-to-volume ratio of 1:7 with an ethanol/water mixture at 10% for 10 min [88]. In a separate study involving the UAE of polyphenols and flavonoids (utilizing a full factorial design of 2^3^, RSM, and quadratic models with R^2^ ranging from 0.77 to 0.98) from various mango by-products, such as peel (1814 and 1228 mg/100 g, respectively), kernel (469 and 653 mg/100 g, respectively), and endocarp (672 and 880 mg/100 g, respectively), the best results were obtained when operating at the central experimental points, with a liquid-to-solvent ratio of 50%, amplitude of 60%, and an extraction time of 20 min. The most significant effects were attributed to the liquid-to-solid ratio and extraction time. In this context, the correct mixture of solvent and solid can expedite the release of BC from plant-based sources. Additionally, all extracts exhibited antioxidant properties, as demonstrated by DPPH tests [97]. Similar trends were observed in the extraction of phenolic compounds from strawberry-guava leaves during UAE (using an FFD of 2^3^, RSM, and a quadratic model with R^2^ of 0.93), where ultrasound power and the solid-to-liquid ratio significantly influenced the extraction yields [37].

In addition, FFD, particularly two-level factorial designs, are commonly employed for the initial assessment of extraction processes, as illustrated in the case of the UAE of phenolic compounds (total phenolics and flavonoids) from malagueta peppers within a multi-response framework [98]. Furthermore, two-level factorial designs are often used to approach an optimal region for further optimization, which can be accomplished through other robust DOEs such as Box–Behnken [99] or central composite designs [101].

Based on these data, FFD could serve as a powerful tool for optimizing the UAE process for the extraction of BC from plant-based materials [97,102]. However, the primary limitation of this type of DOE lies in the number of factors and levels; as they increase, the number of experimental runs increases dramatically, making it a costly and time-consuming method [66].

#### 4.2.1. Fractional Factorial Design

As mentioned earlier, the primary limitation of full factorial designs (FFD) is the substantial increase in the number of experiments as the number of factors increases (number of experiments = Levels × Factors). In this scenario, one alternative for reducing the number of experiments when dealing with an increasing number of factors in FFD is to employ fractional factorial designs [95]. Generally, a fractional factorial design represents a portion (typically ½ or ¼) of the corresponding FFD, allowing for the identification of the main effects of experimental conditions. Fractional factorial designs are particularly valuable in the initial stages of research involving multiple factors, where it is cost-effective to eliminate non-critical conditions in the UAE process before conducting a more comprehensive study with the significant factors [103]. This type of DOE is recommended when the number of factors exceeds 4 [100] and is expressed as 2*^k^*^−*m*^, where “*m*” represents the number of factors that have been removed in the fractional design to reduce the number of experiments [95].

The fractional factorial design has been explored to assess the impact of several factors and levels in UAE processes aimed at recovering BC from plant-based agro-industry foods (Table 3).

Fractional factorial designs serve as valuable screening tools for subsequent optimization using other robust DOEs, particularly when extracting BC from plant-based materials [90]. In this context, it has been observed that the solvent ratio had a significant impact on the UAE of total phenols, flavonoids, and tannins from *Pistacia lentiscus* leaves (2^4−1^ fractional factorial design, RSM, and quadratic model R^2^ ranging from 0.89 to 0.95). Lower ethanol concentrations led to higher yields since the addition of water to ethanol facilitated the extraction of phenolic compounds by breaking hydrogen bonds. However, these results were further fine-tuned using a Box-Behnken design and RSM [90]. Nonetheless, during the UAE of terpenes from *Annona glabra* leaves, (2^7−3^ fractional factorial design), temperature (5 °C) and solvent volume (25 mL) emerged as the most influential factors in terpene recovery, given their pesticidal activities. When it comes to the UAE of polyphenols, flavonoids, and anthocyanins from leaf extracts of *Cecropia* species (2^7−3^ fractional factorial design, RSM, and linear model R^2^ = 0.99), methanol concentration and extraction temperature played pivotal roles in the extraction of these three BC. Furthermore, these results were fine-tuned using a central composite design and RSM [81].

Furthermore, it has been reported that temperature, liquid-to-solid ratio, and ethanol concentration had a significant impact on the UAE of phenols, flavonoids, and anthocyanins from dried sour cherries in a preliminary screening experiment (2^5−1^ fractional factorial design and Pareto chart). These extraction parameters were subsequently fine-tuned using a face-centered central composite design and RSM [106]. Similarly, ethanol concentration and solid-to-liquid ratio played a significant role in the UAE of betulin from white birch bark (2^5−1^ fractional factorial design), with further optimization carried out using a CCD and RSM [103]. Additionally, the liquid-to-solid ratio and extraction time significantly influenced the UAE of phenolic compounds with antioxidant properties from *Moringa peregrina* (2^4−1^ fractional factorial design), with optimization conducted using a central composite design and RSM [105].

These findings highlight the effectiveness of fractional factorial designs as a statistical tool for reducing the number of experiments associated with FFD. Fractional factorial designs are commonly employed as screening tools to identify the most influential parameters affecting the UAE yield of BC from plant-based sources before proceeding to more robust design of experiments.

#### 4.2.2. Plackett–Burman Design

The Plackett-Burman design (PBD) is a two-level factorial design (L12 and L32) developed by Plackett and Burman in 1946. It is designed for studying *K* = *N* − 1 variables in *N* runs, where *N* is a multiple of 4. This design, while not geometrical or regular (meaning it cannot be represented as cubes), serves as a useful tool for initiating the optimization process by screening a substantial number of factors (*X_i_*, *i* > 4) that may influence the response variable *Y*. It achieves this with a relatively low number of experimental runs [66]. One of the key advantages of this design is its ability to eliminate non-significant variables from the models, allowing the selection of important factors for further optimization using other statistical designs such as Box-Behnken or central composite designs [66,73]. In the PBD, variables are assigned two levels (−1 or +1) in coded form, corresponding to two levels of natural variables. Additionally, this DOE includes definitive screening designs (L17/L21) and the folded PBD (L48) [66]. The impact of factors is assessed through a first polynomial equation, with the effect *β_i_* having the highest absolute value corresponding to the most influential factor *X_i_* [64]. It is worth noting that this DOE primarily focuses on the main effects of variables and does not account for interactions [91], as illustrated in Equation (3).

(3) $$Y=\beta_{0}+\sum \beta_{i}\;X_{i}$$

The PBD has been employed to assess the impact of several factors and their levels in the UAE processes for recovering BC from plant-based sources (Table 4).

During the initial optimization of UAE for flavonoids from hawthorn seeds, an L15 PBD ultrasonic time, ethanol concentration, and extraction temperature as the most influential variables. These variables were then chosen for further fine-tuning through Box-Behnken design and RSM [107]. Similarly, when assessing the UAE process for anthocyanins from *Lonicera caerulea* using an L15 PPD, the most significant variables during the preliminary examination were found to be liquid-to-solid ratio, solvent concentration, and extraction time. Subsequently, Box- Behnken design and RSM were employed as robust tools for further optimization [91].

In the case of extracting phenolic compounds with antioxidant properties from grape pomace (L11 PBD), the ethanol concentration and sample-to-solvent ratio had a significant impact on the extraction yield. Notably, the solid-to-liquid ratio had a less pronounced effect on the extraction yield than the ethanol concentration. This can be attributed to the influence of solution polarity due to the presence of water in the extraction solvent, which enhanced the release of water-soluble phytochemicals. These parameters were chosen for further optimization using a 2^3^-full factorial central composite face design [108]. Similarly, in the extraction of polyphenols from rice Caro pods (L11 PBD), particle size was found to affect the process significantly. Reduction in particle size typically leads to an increased contact area, enhancing the cavitation effect. These factors were also selected for further optimization using a Central composite design and RSM [73].

Additionally, the ethanol concentration, extraction temperature, and pH emerged as the most key factors for the recovery of anthocyanin compounds, while ethanol concentration, liquid-to-solid ratio, extraction temperature, and pH played crucial roles in the extraction of phenolic compounds from Rubia sylvatica Nakai fruit (L12 PBD and Pareto chart). These compounds exhibited antioxidant capacity. The UAE process factors related to the phenolic yield were further selected for optimization using a Box- Behnken design and RSM [109]. Furthermore, it has been reported that during the screening step (L12 PBD and Pareto chart) of the UAE for methoxyflavone from *Kaempferia parviflora* rhizomes, the most influential factors included ethanol concentration, solvent-to-solid ratio, and extraction time. These factors were subsequently chosen for further optimization using a Box–Behnken design and RSM [71]. Moreover, the PBD (L12) has been applied to optimize the UAE process for obtaining BC from *Porphyra haitanensis* [110] and *Acer saccharum* [72].

According to these data, the Plackett-Burman design is a viable screening tool for assessing the effects of various variables during the UAE process. It enables the elimination of non-significant variables from the models. After the initial screening, the key factors can be selected for further optimization using a robust DOE.

### 4.3. Box-Behnken Design

The Box-Behnken design (BBD) is an independent rotatory design that employs the midpoints of the edges and center points within the cubic design region. This approach helps in avoiding extreme experimental conditions and reduces the risk of obtaining misleading results [111]. BBD is commonly used for the UAE process due to its efficiency, especially when dealing with three or more variables. It allows for monitoring the independent effects or interactions of these factors on the response variable, all while reducing the number of experiments, time, and costs [92]. Furthermore, BBD is strongly associated with the response surface methodology as a statistical tool applied for optimizing the UAE process [66]. In general, the significance of the factors and their interactions is analyzed based on the following model (Equation (4)):

(4) $$Y=\beta_{0}+\overset{Ε}{\underset{i=A}{\sum }}\beta_{i}\;X_{i}+\overset{Ε}{\underset{i=A}{\sum }}\overset{Ε}{\underset{j=A\neq i}{\sum }}\beta_{ij}\;X_{i}+\varepsilon$$

In this equation, *Y* represents the predicted response, *X_i_* corresponds to the values of the studied factors, *β*_0_ is a constant, *β_i_* stands for the main effect coefficients for each variable, *β_ij_* represents the coefficients for interaction effects, and *ε* accounts for the residual error.

The BBD has been investigated to evaluate the effect of varied factors and levels during UAE processes for recovering BC from plant-based sources (Table 5).

During the UAE of phenolic compounds from *Piper betle* (3^3^ BBD, RSM, and quadratic model R^2^ = 0.98) in a multiple response approach (desirability function), the solid-to-liquid ratio positively influenced the extraction yield. The highest extraction yield was observed at a solid-to-liquid ratio of 1:20 g/mL, accompanied by noticeable antioxidant capacity as determined by DPPH. This effect was associated with a larger concentration gradient of ethanol, which facilitated the propagation of acoustic waves and cavitation. As a result, there was an increase in mass transfer within the system due to enhanced diffusivity [92]. Furthermore, in another multiple response approach, it has been reported that the interaction between time and solvent concentration (3^3^ BBD, RSM, quadratic models, and desirability ranging from 0.94 to 0.97) significantly influenced the UAE yield recovery of phenolics and flavonoids from yellow (R^2^ = 0.95) and red (R^2^ = 0.97) tamarillo fruits [112]. In this case, longer extraction times negatively affected the recovery yield and the antioxidant properties of the compounds. It has been observed that temperature played a facilitating role in the extraction of BC, leading to an increased yield recovery. This increase was attributed to the fact that elevated temperatures improved the solvent’s efficacy due to reduced viscosity and surface tension, allowing for better solvent penetration [98].

In the case of the UAE of phenolic compounds from *Psidium cattleianum* leaves (3^3^ BBD, RSM, and quadratic model R^2^ = 0.99), higher yields were obtained at shorter times, specifically 2 min. Controlling the extraction time in this manner prevented phenolic degradation and preserved their antioxidant properties [31]. Conversely, during the UAE of phenolic compounds from muicle leaves (3^3^ BBD, RSM, and quadratic model R^2^ = 0.97), it was observed that all factors significantly influenced the extraction process, with pulse cycle duration having the most substantial effect. The pulse cycle’s duration was associated with an increase in microbubble formation, which enhanced cell damage and facilitated the higher release of phenols. These phenols exhibited good antioxidant properties in various in vitro tests, including DPPH, ABTS, and FRAP [113]. It is worth noting that the efficiency of the UAE process, specifically pulse cycle, amplitude, and time, depends on the plant matrix’s composition (cellulose-hemicellulose-lignin ratio) and experimental conditions. This was evident during the UAE of phenolic compounds from various parts of *Annona muricata* by-products, such as pulp, peel, seeds, and columella. Each of these materials was studied using a BBD, RSM, and quadratic models, and the results showed R^2^ values of 0.92, 0.98, 0.97, and 0.91, respectively [114]. Similar trends were reported by González-Peredo et al. [111], who found that the recovery yield of BC from myrtle using UAE depended on the type of compounds. However, for phenolics, they noted that the interaction between solvent and temperature, as well as the interaction between cycle and liquid-to-solid ratio, had significant effects on phenolic yield. Moreover, solvent and pH were significant factors affecting anthocyanins yield (3^6^ BBD, RSM, and quadratic models R^2^ = 0.99).

Additionally, it has been reported that using 30% *v*/*v* ethanol with a liquid-to-solid ratio of 5 mL/g at 55 °C for 25 min is an effective method for increasing the extraction yield of BC from common century under UAE (3^4^ BBD, Pareto chart, and quadratic model R^2^ = 0.99). However, it is important to note that all individual and interacting parameters significantly influenced the extraction process [4]. Furthermore, it has been reported that solvent concentration plays the most crucial role (along with temperature, amplitude, cycle, pH, and liquid-to-solid ratio) in the UAE of phenolic compounds and anthocyanins from jabuticaba fruits. This was determined using a 3^6^ BBD, RSM, Pareto chart, and quadratic models, with R^2^ values of 0.70 and 0.82, emphasizing the significance of solvent concentration in the extraction process [57].

On the other hand, in pomegranate peels [115], it was observed that the methanol concentration and amplitude had a significant impact on the UAE yield recovery of phenolic compounds. In contrast, for flavonoids (3^4^ BBD, RSM, and quadratic model R^2^ = 0.84–0.90), the pulse cycle and amplitude were found to be significant factors. The optimal multi-response conditions to maximize the total phenolic and flavonoid contents, along with higher antioxidant capacities assessed by DPPH and FRAP, were determined as follows: 70% methanol, 80% ultrasound amplitude, 0.58 s pulse cycle, and 12.8 min of extraction time. Furthermore, BBD, when applied in a multiple response approach using the desirability function, has been effectively used to optimize preliminary results. These preliminary results were obtained from other DOEs such as simplex-centroid or two-level factorial design, and they affected the analytical response during the UAE of phenols and flavonoids from malagueta peppers [98].

According to these data, it is evident that several factors can influence the efficiency of the UAE process. Therefore, BBD is a valuable experimental design that allows for the evaluation of factors and their interactions in a reduced number of experiments. Moreover, BBD can be effectively employed in conjunction with other DOEs.

### 4.4. Central Composite Design

The central composite design (CCD) is one of the most widely used statistical tools in optimization. It offers great flexibility and can be constructed from a 2*^k^* full factorial design or 2*^k^*^−*p*^ factional design, with the addition of axial and central points. This type of DOE can accommodate a full quadratic model [66], as demonstrated in Equation (5).

(5) $$Y=\beta_{0}+\overset{Ε}{\underset{i=1}{\sum }}\beta_{i}\;X_{i}+\overset{Ε}{\underset{i=1}{\sum }}\beta_{ii}X_{i}^{2}\overset{Ε}{\underset{j=j\neq 1}{\sum }}\beta_{ij}\;X_{i}\;X_{j}\;$$

where *Y* is the predicted response, *X_i_* and *X_j_* are the levels for the studied factors, *β*_0_ is a constant, *β_i_*, *β_ii_*, and *β_ij_* are the main effect coefficients for each variable, and *β_ij_* are the interaction effect coefficients.

The CCD has been investigated to evaluate the effect of different factors and levels during UAE processes for recovering BC from plant-based sources (Table 6).

According to the literature, the most common form of the CCD includes three independent variables with five levels. In the context of UAE (using a CCD with three factors and five levels, combined with RSM and quadratic models with R^2^ values ranging from 0.90 to 0.94 and desirability of 0.82), the extraction of bioactive phenolic and flavonoid compounds with antioxidant properties from spinach roots was investigated in a multiple response approach [2]. The results showed that the highest antioxidant capacity was achieved at 40% amplitude and 40 °C for 4 min. Notably, ethanol concentration emerged as the most critical factor influencing the extraction process. Higher ethanol concentrations led to decreased extraction yields of phenolic and flavonoid compounds, affecting their antioxidant capacity. This phenomenon is likely due to the greater solubility of BC in spinach roots in water than in ethanol. Similar trends were observed during the extraction of phenolic compounds from black locust flowers using ultrasound (employing a CCD with three independent variables at five levels along with RSM and quadratic models with R^2^ values of 0.94 and a desirability function) in a multiple response approach. The highest extraction yield was achieved with a 60% ethanol concentration for 30 min at 59 °C. However, the impact of ethanol concentration on the response depended on the extraction time, with the solvent’s effect being negligible at shorter times [84]. Furthermore, it has been reported that the optimal conditions for obtaining compounds with desired bioactivity, such as skin whitening and anti-wrinkle effects, were a temperature of 52.1 °C and an ethanol concentration of 50.7% for 26.4 min during UAE from safflower seeds (utilizing a CCD with three independent variables at five levels, combined with RSM, and quadratic models with R^2^ values ranging from 0.88 to 0.93). Ethanol concentration was identified as the most significant factor associated with the polarity of the extractable compounds. Similarly, the highest UAE yield of total phenolic compounds from wheatgrass seeds was achieved using a CCD with three independent variables at five levels, RSM, a quadratic model with an R^2^ of 0.99, and a desirability function. The optimal conditions included a 56% ethanol concentration, 28 min at 59 °C [120].

In the context of UAE (utilizing a CCD with two independent variables at five levels, combined with RSM, quadratic models with R^2^ values of 0.80 and desirability of 0.74) for extracting phenolic compounds from chestnut shells, it was found that temperature and extraction time significantly influenced the extraction yield. The highest antioxidant activities were observed when the process was conducted at 70 °C for 40 min. This suggests that, under these specific experimental conditions, a greater quantity of polyphenols with antioxidant properties are extracted. However, it is important to note that while increasing the temperature may lead to higher yields of polyphenols, it also carries the risk of compound degradation during the extraction process [117]. On the other hand, the highest extraction yield of phenolic compounds from *Sideritis raeseri* using UAE (employing a CCD with four independent variables at five levels, RSM, quadratic models with an R^2^ of 0.81, and a multiple response approach) was achieved at 65% ethanol concentration for 50 min at 63 °C, with a solid-to-liquid ratio of 1:40. It’s worth noting that the extraction temperature and the solid-to-liquid ratio had a significant impact on the recovery yield of compounds. Interestingly, no interactions between the factors were observed, and they did not influence the response [119].

Compared to CCD, a central composite rotatable design (CCRD) maintains a consistent prediction variance at equally spaced locations from the design center. In the context of UAE (utilizing CCRD, RSM, and quadratic models) for extracting BC with antioxidant properties (total anthocyanins R^2^ = 0.77, carotenoids R^2^ = 0.85, phenolic compounds R^2^ = 0.97, total flavonoids R^2^ = 0.89) from agro-industrial acerola residue, a multiple response approach employing the desirability function revealed the optimal conditions for achieving the highest yield of BC. These conditions involved an ethanol concentration of 46.49%, an ethanol-residue ratio of 8.66 mg/L, and an extraction time of 49.30 min. Notably, increasing the ethanol-residue ratio facilitated the interaction between the solvent and solute, leading to enhanced bioactive compound recovery. Additionally, the extraction time played a crucial role in preserving the antioxidant properties of the extracted compounds [116].

Similarly, it has been reported that the optimal conditions for UAE (utilizing CCRD, RSM, and quadratic models) to achieve the highest yield of BC, including phenolics and flavonoids with antioxidant properties, from *Ficaria kochii* were a solvent-to-raw material ratio of 90:10, an extraction time of 30 min, and a temperature of 40 °C. These parameters play a crucial role in determining the yield of phenolic compounds. It was observed that as these parameters increased, the phenolic content decreased, possibly due to a dampened cavitation effect associated with higher temperatures [51]. In this context, CCRD and RSM have been effectively utilized to optimize the UAE process for extracting BC from a variety of sources, including *Ocimum tenuiflorum* leaves [121], kiwi peel [122], *Myrtus communis* L. pericarp [123], palm pressed fiber [124], green propolis [101], and *Mitragyna speciosa* (Korth.) Havil leaves [125], and garlic leaves [20].

The face-centered design is a type of CCD with an alpha value of 1, in which axial points are positioned at the center of each face in the factorial space. This DOE requires three levels for each element. The Central Composite Face-Centered Design (CCFCD) has been explored to optimize the UAE process for obtaining BC from plant-based materials. For instance, in the case of UAE for BC with antioxidant properties from *Garcinia indica* (CCFCD, RSM, and quadratic models with R^2^ values ranging from 0.70 to 0.73), the extraction yield was found to be influenced by experimental conditions for both BC (total phenolics and total flavonoids) and their antioxidant activities (DPPH, ABTS, and FRAP). Notably, these extracts also exhibited anticancer activity against human breast cancer cells [109]. Additionally, the CCFCD has been employed to optimize the UAE process for extracting various BC from *Nephelium lappaceum* L. fruit peel [69] and *Taraxacum officinale* [126].

Additionally, a non-standard central composite design (NSCCD, RSM, quadratic model with R^2^ > 0.80 and desirability score of 100%) successfully optimized the UAE of polyphenols from *Ceratonia siliqua* L. in a multiple response approach. The most critical factor influencing the recovery yield was the solid-to-liquid ratio [73]. 

Based on these data, the CCD proves to be an effective tool for optimizing the UAE process, offering good predictive capabilities. It can also be employed in conjunction with other experimental designs and a variety of statistical tools, including RSM.

### 4.5. Taguchi Design

Taguchi design (TD) is a unique and powerful screening tool for identifying the significant factors that affect the extraction, both in terms of quantity and quality, of BC from natural sources in multifactorial experiments with mixed levels [70]. It is a robust parameter design to optimize UAE processes, thereby reducing the number of experiments, time, and costs [127]. Its effectiveness stems from its capability to distribute factor levels in a well-balanced manner, minimizing process variation [66,128]. The Taguchi approach involves arranging experiments based on an orthogonal array index. The size of this array depends on the total parameters and their respective settings. To conduct a partial factorial experiment effectively, a certain number of degrees of freedom (*DOF*) or minimum experiments are needed to represent the system accurately [129]. This minimum experiment number or *DOF* is defined in Equation (6):

(6) $$DOF=1+\overset{n}{\underset{i=1}{\sum }}\left(Ni-1\right)\;$$

where *n* and *Ni* denote the number of experiments and parameter levels, respectively.

In the TD, the outcomes of experiments are transformed into a signal-to-noise (S/N) ratio and are represented in decibels (dB). Three various signal-to-noise ratios are available, including “smaller-the-better”, (minimize a non-negative characteristic value, *Y_i_*) “larger-the-better”, (maximize a non-negative characteristic value, *Y_i_*) and “nominal-the-better”, (minimize the variation between the characteristic value, *Y_i_*, and the target value, *Y*_0_) which depend on the preferred mean square deviation (*MSD*). The *MSD* represents the average performance characteristic value for each experiment (Equations (7)–(9)). The distinct signal-to-noise ratios (η) for *n* experiments are shown in Equation (10):

Smaller-the-better

(7) $$MSD=\frac{1}{n}\;\overset{n}{\underset{i=1}{\sum }}{\left(Y_{i}\right)}^{2}$$

Larger-the-better

(8) $$MSD=\frac{1}{n}\;\overset{n}{\underset{i=1}{\sum }}{\left(\frac{1}{Y_{i}}\right)}^{2}$$

Nominal-the-better

(9) $$MSD=\frac{1}{n}\;\overset{n}{\underset{i=1}{\sum }}{\left(Y_{i}-Y_{0}\right)}^{2}$$

Signal-to-Noise Ratio

(10) $$\eta =-10.0\;log10\left(MSD\right)\;\;$$

TD has been utilized in the optimization of processes, including UAE of polyphenols from plant-based sources, as listed in Table 7.

The application of TD facilitates the identification of the optimal quantity of experiments required to yield the most advantageous data for a given set of factors [70]. During the UAE (L9 TD) of anthocyanins from red cabbage, the time, temperature, and power ultrasound were the factors that positively contributed to the UAE efficiency, where the optimum condition was at 15 °C and 100 W for 30 min. In this study, ultrasound facilitates the release of anthocyanins from red cabbage [70]. On the other hand, the high-yield extraction of anthocyanins and phenolic compounds from butterfly pea petals by UAE (L9 TD, RSM, and quadratic model) was under 40 °C at 10 mL/g for 45 min of extraction; however, the liquid: solid ratio significantly contribute to the yield recovering of anthocyanins and phenolic compounds, while high temperatures resulted in a decrease in yield [24]. Similar trends were reported in the UAE of polyphenols with antioxidant properties from *Hamelia patents* (L9 TD), where the liquid: solid ratio is the main factor influencing the UAE processes. Thus, a correct solvent-solid mixture can accelerate the release of BC from plant-based sources [131]. An L9 Taguchi orthogonal design has also been used to optimize the UAE of curcumin from *Curcuma longa* rhizomes [130].

In the context of UAE (using an L16, signal-to-noise ratio as larger is better) of phenolic compounds from *Azadirachta indica*, tit was found that the particle size significantly influenced the extraction yield, followed by temperature and time. In this context, a reduction in the size of particles typically increases the contact area between plant cell walls and ultrasound, enhancing the yield extraction of BC; moreover, this fact is improved by temperature, which increases the mass transfer [76]. On the other hand, the optimal UAE (L27 TD, signal-to-noise ratio as larger is better) of anthocyanins from *Clitoria ternatea* petals were at 50 °C and 10:1 mL/g for 30 min [132]. Additionally, the liquid: solid ratio, ethanol concentration, and extraction temperature were the most critical factors that affected the recovery yield of polyphenols from coffee leaves under UAE in a multiple response approach (L8, RSM, quadratic models R^2^ of 0.91, and desirability of 0.95); these factors were selected for further optimization using a BBD and RSM [127].

The TD is commonly regarded as superior to traditional factorial or fractional factorial designs due to its ability to extract more accurate information from a limited number of experimental runs [130]. According to these data, TD is a helpful tool for screening and optimizing the UAE process, which can offer a reduced number of experiments and demonstrate the main effects in a multifactorial experiment for further optimization using a robust DOE.

### 4.6. Mixture Designs

During the UAE of BC from plant-based materials, solvent viscosity, polarity, vapor pressure, and surface tension play a crucial role in the efficiency of the extraction procedure [132]; therefore, the solvents can be used alone or in mixture solutions [133,134]. In the case of mixture solutions, it is necessary to identify the most suitable combination of solvents for extracting BC without affecting their biological properties [135]. In this context, mixture designs are widely used to address formulations, where simplex designs are the simplest that describe mixtures in proportions ranging from 0 to 100% [100], in binary, ternary, and or even multi-component solvent mixtures [133]. The Simplex-Centroid design (SCD) also allows observing synergistic/antagonistic effects between solvents, influencing the recovery yield of BC. In a typical scenario involving mixture experiments, there are *q* components or ingredients, and each experimental treatment is based on a specific blend or mixture of these ingredients. If we represent these proportions as *x*1, *x*2, …, *xq*, the components’ participation must adhere to two specific constraints: (i) the proportions must be values between zero and one; (ii) the *q* proportions always add up to one, resulting in the levels of the components *X_i_* not being independent of each other [133,134,135].

Once the experimental results from a mixture design are available, it is essential to establish a statistical model to explore the impact of the components on the response variable. A first approach would be to fit a first-order model (Equation (11)). On the other hand, the quadratic model for *q* components is provided by Equation (12):

(11) $$E\left(y\right)=\beta_{0}+\overset{q}{\underset{i=1}{\sum }}\beta_{i}X_{i}$$

where, *E*(*y*) is the expected value of the response variable.

(12) $$E\left(y\right)=\overset{q}{\underset{i=1}{\sum }}\beta_{i}X_{i}+\sum \overset{q}{\underset{i<j\;j=2}{\sum }}\beta_{ij}X_{i}X_{j}$$

where the coefficient *β_i_* represents the expected response in the pure mixture *X_i_* = 1, and at the same time, it is the height of the surface at the vertex *X_i_* = 1.

In general, the special cubic model for *q* components is given in Equation (13):

(13) $$E\left(y\right)=\overset{q}{\underset{i=1}{\sum }}\beta_{1}X_{i}+\sum \overset{q}{\underset{i<j\;j=2}{\sum }}\beta_{ij}X_{i}X_{j}+\underset{i<j}{\sum }\underset{j<k}{\sum }\overset{q}{\underset{k=3}{\sum }}\beta_{ijk}X_{i}X_{j}X_{k}$$

The SCD and Lattice-Simplex design (LSD) have been employed to define the best extraction solvent mixture for optimizing UAE of BC from plant-based sources, as shown in Table 8. 

It is well-known that the polarity and viscosity of the solvent can influence the ultrasound-assisted extractability of BC from plant materials. Therefore, a mixture of extractor solvents is frequently used during the UAE process [136]. In this context, Moreira et al. [136] optimized the extractor solvent for recovering phenolic compounds by UAE from *Physalis angulate* throughout a simplex-centroid design (SCD, RSM, and quadratic models R^2^ = 0.99). They reported that the highest polyphenol yield was obtained with a solvent mixture composed of 57% water, 35% ethanol, and 8% methanol with a time of 10 min and 15 mL of extractor volume. These results were associated with the low viscosity of the solvent mixture that significantly enhanced the cavitation process. Similar trends were reported during the UAE (SCD, RSM, quadratic models R^2^ of 0.97–0.99, and desirability of 0.99) of polyphenols and flavonoids from Moroccan *Pimpinella anisum* in a multiple response approach, where the best proportions of extractor solvent were 44% water, 22% ethanol, and 34% methanol [135].

Additionally, it has been reported that the ethanol-water (68:32) mixture is effective in increasing the UAE yield of polyphenols from *Taraxacum assemanii* (SCD, RSM, and quadratic model R^2^ = 0.90–0.93); moreover, in this study, the interactive effect between ethanol and methanol negatively affected the recovery yield polyphenols, possibly by the polarity of bioactive compound of *T. assemanii* [133]. On the other hand, during the UAE (SCD, RSM, Pareto chart, quadratic model, and desirability function) of polyphenols from *Capsicum frutescens*, the best mixture proportion of extractor solvent was 95% ethanol and 5% water [98]. Ethanol is a commonly used solvent for phenolic extraction in plants, but other solvents such as methanol, water, or a mixture of both are recommended for extracting phenolic acids [21]. Additionally, it has been reported that combining ethanol and acid solution (1 mol L^−1^ HCl) at 50:50 significantly increased the UAE (SCD, RSM, and quadratic model R^2^ = 0.98) of polyphenols from pineapple byproducts. These effects were associated with the membrane denaturation effect of HCl [138].

By combining 46% water, 13% methanol, 18% ethanol, and 23% acetone, it is possible to increase the UAE (SCD, RSM, quadratic model R^2^ = 0.94, and desirability function) yield of polyphenols from *Eugenia uniflora* [130]. In mango peels, the best proportion of extractor solvent for increasing the UAE polyphenol yield was 60% ethanol and 40% acetone [74]. It has been reported that combining polar-protic and polar-aprotic solvents (ethanol-acetone) mixture is more effective than pure solvents, associated with their polarity and the solubility of phenolic compounds, and the ability of aprotic solvents to solvate compounds with the low and high molecular weight with protonatable functional groups, while polar-protonic solvents as ethanol that contains hydroxyl groups (hydrogen donor) that facilitate the extraction of low molecular weight compounds [74].

The best combination of extractor solution for recovery of the maximum yield of carotenoids from cashew apple by UAE (SCD, RSM, and quadratic model R^2^ = 0.98) was 44% acetone and 56% methanol. The resulting mixture had an intermediate polarity that increased the solute-solvent interaction and improved the dissolution and extraction of carotenoids. Interesting, the ternary mixtures composed of ethanol/petroleum ether/methanol and acetone/petroleum ether/methanol exhibited antagonistic effects during the UAE of carotenoids (SCD, RSM, and quadratic model R^2^ = 0.93–0.95) [137]. 

In a multiple response approach (LSD, RSM, and a special cubic model R^2^ = 0.98 and desirability of 0.93), the best proportions of solvents for UAE of carotenoids from *Mauritia flexuosa* were 75:25 acetone: ethanol, which exhibited a synergistic effect during the extraction process. On the other hand, mixtures of acetone/methanol and acetone/acetonitrile exhibited antagonistic effects. It is attributed to the non-polar compounds of *M. flexuosa*, and the mixture of acetone/methanol efficiently extracted the BC of this plant-based material [134].

According to these data, mixture designs, mainly the SCD, are an effective optimization technique for selecting the most appropriate mixture solvent to extract BC by UAE.

### 4.7. D-Optimal Design

D-optimal design (DOD) is characterized by an orthogonal array (but not exclusively). It is recommended for performing screening and optimizing experiments. DOD can minimize the number of runs and the variance in the prediction of specified model coefficients and is compatible with the RSM [140,141,142]. This design of experiments was created to allow the study of multiple combinations of multilevel factors, independently if the number of variable levels of factors is different in the same experimental design [86,143,144]. DOD has been investigated as a statistical tool for optimizing the UAE of BC from plant-based sources (Table 9).

In the context of UAE of phenolic compounds and flavonoids from walnut male flowers using a DOD (RSM and quadratic model R^2^ = 0.95 and 0.93, respectively), it was found that the most important factor that influenced the extraction yield of polyphenols was the content of water in the solvent followed by the type of solvent used, where the higher extraction yield was performed after 30 min of extraction containing 40% water in acetone. These effects were associated with the polar aprotic and polar protic characteristics of extractor solvent, which is more efficient than the pure solvents or the mixture of two polar protic solvents [87]. Similar trends were reported during the UAE (DOD, RSM, quadratic model R^2^ of 0.99, and desirability function) of phenolic compounds from grapefruit leaves, where the best UAE conditions include ethanol concentration of 10.80% at 30.37 °C for 58.52 min. In this study, the presence of higher content of water in the extractor solutions positively affect the recovering yield of polyphenols, associated with its polarity, dielectric constant, and viscosity that help the swelling increase of the cell, releasing BC [141].

Furthermore, it has been reported that the optimal conditions for extracting phenolic compound from *Echinacea purpurea* by UAE (DOD, RSM, quadratic model R^2^ of 0.96 and desirability of 0.98) were using 41.70% methanol at 75 °C for 51.8 min. In this study, the temperature and ultrasound waves combined with methanol facilitate the release of BC from the plant matrix; however, these conditions should be further optimized with a robust DOE to avoid their degradation during UAE [144]. 

In the case of the UAE of phenolic and flavonoid compounds from *Cynara* *scolymus* leaves (DOS, RSM, quadratic model R^2^ > 0.95, and desirability function), the best conditions for obtaining a maximum yield of BC from this plant material were 20.05 min of extraction time and 65.02% of ultrasound amplitude, where the ultrasound amplitude was the most important variable [145]. Moreover, it has been reported that time (5 min), amplitude (30%), and ethanol concentration (50%) significantly influenced the extraction of phenolic compounds from wild thyme and their antioxidant properties, as demonstrated by Babotă et al. [143] during the UAE process (DOD, RSM, and quadratic model R^2^ = 0.95). Additionally, DOD has been used to optimize the AUE of oleuropin from mango peels (desirability of 0.79–0.89) [142].

According to these data, DOD can maximize the determinant of the information matrix, requires a reduced number of runs, and is compatible with RSM. This DOE is typically used to identify the main factors involved in the UAE of BC from plant-based materials.

### 4.8. Doehlert Design

David H. Doehlert created the Doehlert design matrix (DD). It is also known as the Uniform shell design. DD is derived from a spherical domain with an orthogonal array and is a not-rotatable DOE [146]. This design is generated from regular geometric shapes with k + 1 vertices (k-dimensional simplexes), circumventing a central point indicating an optimal region [67]. An important feature of DD is that it enables the examination of multiple variables with different levels within a single matrix, reducing the number of experiments [147]. 

In the case of the UAE of phenolic compounds from *Croton heliotropiifolius* Kunth leaves (DD and Pareto chart), the higher yield was obtained at experimental conditions of 11.4 mL of 37.5% v/v ethanol at a temperature of 54.8 °C for 39.5 min [147]. Additionally, DD was used to find the ideal values for the extractor solution volume and sonication time for the UAE of polyphenols from *Physalis angulate* L. by Moreira et al. [136], who informed that the best conditions for the extractor solvent was the ratio of 57:35:8 in water: ethanol: methanol. Furthermore, the best condition for sonication time was 10 min, using 15 mL of extractor volume. Also, this DOE has been investigated to optimize the time, temperature, and power ultrasound conditions during the UAE of essential elements (Ca, Mg, K, P, S, Fe, Cu, and Mn) in guarana samples [146]. 

The DD is typically used for optimizing several analytical procedures and has recently been explored to optimize the UAE process for obtaining BC from plant materials [136,147].

### 4.9. Combined Designs

DOEs are typically investigated to optimize processes, including the UAE of BC from plant-based materials [64]. Although the extraction process typically applies a single DOE, it can be investigated using combined DOEs for screening and optimizing the UAE processes [81,90,113,148]. On the other hand, due to the lack of guidelines and suggestions about how combining different designs can influence the outcomes of analysis using these techniques, further studies are needed to address the knowledge gap on combining DOEs to optimize UAE processes to maximize the recovery yield of BC from plant-based materials [66]. Table 10 lists some studies where combined DOEs were applied to optimize the AUE of BC from plant-based materials.

## 5. Recommendations to Select an Analytical DOE: Advantages and Limitations

As mentioned before, the efficiency of the UAE depends on many factors. Therefore, selecting an appropriate DOE is required. In general, the statistical procedures depend on the research aims; nonetheless, experimenters should pose some questions before deciding on a DOE such as the following:

1. How many independent variables (factors) and levels per variable are there?
2. How many experimental runs will be there?
3. Is it a screening or an optimizing experiment?

**Figure 1:**
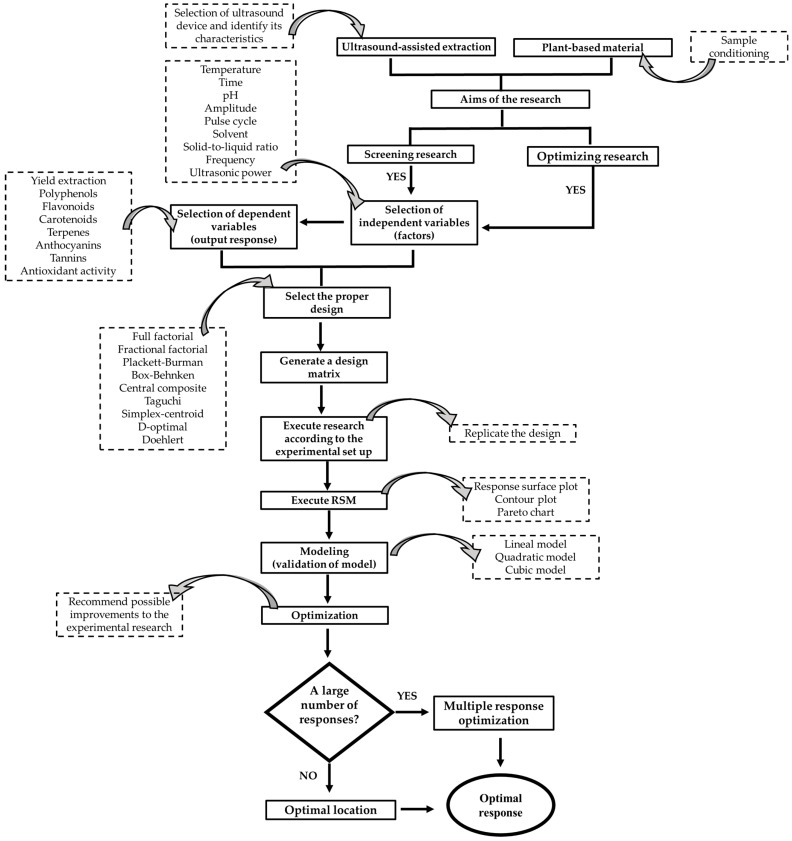
Schematic representation of the design of experiments applied during ultrasound-assisted extraction of bioactive compounds from plant-based materials (Adapted from [68,83,149]).

A proposed diagram based on the information discussed throughout this work is shown in Figure 1. It includes a global view for optimizing UAE processes to recover BC from plant-based materials using DOEs as a statistical strategy. Briefly, in the pursuit of optimizing the extraction of BC through ultrasound-assisted techniques, the research objectives are meticulously defined to outline the overarching goals unequivocally. This involves the identification of key factors, including ultrasound frequency and power, and subsequent determining optimal extraction levels. The selection of plant material is a nuanced process, intricately considering BC content and availability to ensure a judicious choice of representative samples. The decision-making process extends to determining the number of experimental runs, where considerations of ultrasound time and biological variability of the plant material come to the forefront. The experimental type is explicitly specified by choosing between screening or optimization, adeptly factoring in the inherent variability of the selected plant material. The selection of an experimental design is a pivotal step, seamlessly integrating ultrasound parameters and plant material characteristics. Implementation of the experiment with ultrasound is executed based on the intricacies of the selected design. Subsequent data analysis considers the influence of ultrasound technology and plant material characteristics, facilitating a comprehensive assessment. Effectiveness is gauged through a comparative analysis of different designs, considering both ultrasound factors and plant material characteristics. Goal attainment is evaluated by scrutinizing the achievement of research objectives, considering both ultrasound-assisted extraction and plant material selection. The culmination of this meticulous process is marked by the recognition of the study’s endpoint.

Additionally, it is important to note that each DOE exhibits its advantages and limitations, as shown in Table 11.

## 6. Conclusions

Ultrasound-assisted extraction is a green technology with competitive advantages to recover bioactive compounds from plant-based sources with higher yields than conventional extraction methods. However, the parameters involved in the UAE process influence its efficiency in the recovery yield of target compounds and their bioactivities. Thus, optimizing UAE conditions is necessary to achieve higher quality and yield bioactive compounds since the extraction conditions are dependent on the characteristics of each plant-based matrix.

Design of experiments (DOEs) plays an essential role during the ultrasound-assisted extraction of bioactive compounds from plant-based sources. It permits the identification of the kay variables or factors involved in the extraction process, which facilitates the optimization of UAE in a single or multi-response approach. The main DOEs investigated for screening and optimizing UAE processes are Full factorial, Fractional factorial, Plackett–Burman, Box-Behnken, Central composite, Taguchi, Simplex-centroid and Lattice-simplex, D-optimal, and Doehlert. Most of these statistical designs are optimized using response surface methodology and polynomial equations that permit the prediction and validation of response variables.

For its part, Derringer’s desirability function is a viable option when several response variables should be simultaneously optimized. Additionally, the UAE of bioactive compounds from plant-based materials could be performed in a combined DOE performance, one of them focused on the screening step and the other one on optimizing.

In this context, during the optimal DOE selection, the physical limitations of experiments (time, material resources, and the ability to perform experiments under extreme conditions) must be considered. The selected DOE should be a secure image of interactions with the minimum amount of resources possible during the conduction of experiments. Therefore, this document provides a general overview of the most used DOE (alone or combined) for UAE of bioactive compounds from plant-based sources and how the optimal DOE can be selected depending on the research aim. However, further studies are needed to standardize the extraction conditions independently of the plant-based matrix that permits narrowing the gap between research laboratories and industry scale-up of UAE of BC from plant-based materials.

## Figures and Tables

**Table 1:** Conventional and non-conventional technologies for extracting phenolic compounds from *Psidium cattleianum* leaves.

Extraction Method	Solvent	Extraction Time (min)	Yield (%)	Ref.
Maceration	Ethanol-Water	120	0.31	[32]
Soxhlet	Petroleum ether	360	0.49	[33]
Shaking	Methanol-Acetone-Water	120	6.52	[31]
Hydrodistillation	Water	180	0.40	[33]
Aqueous infusion	Water	10	<0.01	[34]
* Stirring	Methanol	4320	15.72	[35]
Supercritical fluid	CO_2_	180	0.03	[33]
Pressurized fluid	Water	20	0.44	[34]
Enzymatic	Water	360	12.1	[36]
Ultrasound bath	Water	180	10.1	[36]
Ultrasound (sonicator tip)	Hexane	5	2.55	[37]
Ultrasound (sonicator tip)	Methanol-Acetone-Water	4	15.81	[31]

Adapted from González-Silva et al. [31]. * Extract was fractioned and concentrated.

**Table 2:** Factorial designs used for UAE of bioactive compounds from plant sources.

Source	Bioactive Compound	Ultrasonic Equipment	DOE	Single or Multiple Response	Factors and Levels	Number of Runs	Results	Ref.
*Fagus sylvatica* bark	Polyphenols	Ultrasonic bath at 40 kHz of frequency	Factorial 3^3^	Single	Ethanol (50%, 70%, and 100 *v*/*v*), extraction time (15, 30, and 45 min), temperature (50, 60, and 80 °C)	27	Solvent concentration and temperature exhibited significant effects on UAE yield	[86]
Lime, orange, and tangerine peels	Phenolic compounds	Ultrasonic bath at 60 kHz of frequency	Factorial 2^2^	Single	Water content of peel (0 and 75%) and extraction time (30 and 90 min)	Four runs for each citrus peel	Extraction time had no significant effect on UAE yield	[50]
Common bean	Phenolic compounds	Ultrasonic bath	Two-level factorial (2*^k^*)	Single	Extraction time (40 and 80 min), temperature (30 and 50 °C), ultrasonic power (400 and 560 W), liquid-to-solid ratio (30 and 40 mL/g), Acetone concentration (40 and 60%)	16	Extraction time, acetone concentration, and liquid-to-solid ratio were the top three factors that influenced the UAE yield	[96]
Mango by-products (peel, endocarp, and kernel)	Polyphenols and flavonoids	Ultrasonic bath at 80 kHz	Factorial (2^3^) with three central points	Single	Liquid-to-solvent ratio (0, 50, and 100%), amplitude (30, 60, and 90%)	11 runs by each product	Solvent relation and extraction time significantly influenced the UAE yield	[97]
*Nephelium lappaceum* husk	Phenolic compounds	NI	Factorial 3^3^	Single	Solid-to-liquid ratio (1:3, 1:5, and 1:7), extraction time (10, 15, and 20 min), and ethanol concentration (10, 30, and 50%)	27	Solid-to-liquid ratio significantly influenced the UAE process	[88]
Strawberry-guava leaves	Phenolic compounds	Ultrasonic probe at 20 kHz of frequency coupled with a titanium tip of 4 mm diameter	Factorial 2^3^ and central points	Single	Temperature (40, 50, and 60 °C), ultrasonic power (100, 300, and 500 W), and leaf: solvent ratio (1:10, 1:15, and 1:20 g/mL)	11	Power and solid-to-liquid ratio exhibited significant effects in UAE yield	[37]
Malagueta peppers	Phenolics and flavonoids	NI	Factorial (2^3^) with three central points	Multiple	Solvent volume (5 and 15 mL), extraction time (2 and 20 min), temperature (30 and 50 °C)	11	Factorial design was used for the preliminary evaluation of extraction conditions	[98]
Olive pomace	Phenolic compounds	Ultrasonic probe	Factorial (2*^k^*) with five central points	Single	Amplitude (30, 50, and 70%) and extraction time (2, 7, and 12 min)	9	Two-level factorial design was used to reduce optimal extraction time obtained previously form a Box-Behnken design	[99]
Fresh green olive leaves	Phenolic compounds	Ultrasonic bath at 37 kHz of frequency	Factorial	Single	Solvent concentration (20, 50, 70, and 90% *v*/*v*), extraction time (10 to 120 min), and temperature (30 to 65 °C)	15	Solvent concentration and extraction time significantly influenced the UAE process	[22]
Spruce wood bark	Polyphenols	Ultrasonic bath at 35 kHz of frequency and power of 320 W	Complete factorial (3^2^·2) with three central points	Single	Temperature (40, 50, and 60 °C), time (30, 45, and 60 mi), and ethanol concentration (50 and 70% *v*/*v*)	18	Ethanol concentration and extraction time were the most significant factors that improving extraction yield	[89]

NI: No information.

**Table 3:** Fractional factorial designs investigated for UAE of bioactive compounds from plant sources.

Source	Bioactive Compound	Ultrasonic Equipment	DOE	Single or Multiple Response	Factors and Levels	Number of Runs	Results	Ref.
*Annona glabra* leaves	Terpenes	Ultrasonic probe 2- and 7-mm diameter, 24 kHz of frequency and 200 W of power, pulse cycle of 0.1 to 1 s	2^7−3^	Single	Temperature (5 and 25 °C), volume (25 and 50 mL), time (5 and 15 min), probe (2- and 7-mm diameter), solvent (methanol and acetone), amplitude (30 and 70%), cycle (0.2 and 0.8 s)	16	Temperature ad solvent volume were the most notable factors for increasing UAE yield	[104]
*Moringa peregrina*	Phenolic compounds	Ultrasonic bath at 20 kHz of frequency and 580 W of power	2^4−1^	Single	Liquid-to-solid ratio (5 and 15 mL/g), ultrasound power (30 and 100%), time (5 and 25 min), temperature (30 and 60 °C)	8	The liquid-to-solid ratio and extraction time had significant effects on UAE yield	[105]
White birch bark	Betulin	Ultrasonic probe 12.7 mm diameter, 20 kHz of frequency and 450 W of power	2^5−1^	Single	Ethanol concentration (65, 80 and 95% *v*/*v*), solid-to-liquid ratio (1:40, 1:25, and 1:10), extraction temperature (40, 50, and 60 °C), ultrasonic frequency (2, 5. And 8 kHz), extraction time (1, 3, and 5 min)	16	Ethanol concentration and solid-to-liquid ration significantly influenced the UAE yield	[103]
Sour cherries	Total phenolics, flavonoids, and anthocyanins	Ultrasonic bath at 40 kHz frequency	2^5−1^	Single	Temperature 40 and 60 °C, extraction time 20 and 40 min, ethanol concentration 40 and 60% *v*/*v*, ultrasonic power 30 and 60 W/L, and liquid-solid ratio 10 and 20 mg/L	16	Temperature, liquid-to-solid ratio, and ethanol concentration had significant effects on UAE yield	[106]
*Cecropia* species, leaves	Phenols, flavonoids, and anthocyanins	Ultrasonic bath at 42 kHz frequency and 100 W of power	2^7−3^	Single	Methanol concentration (50 and 90%, *v*/*v*), extraction time (30 and 90 min), number of extractions with methanol (1 and 3), extraction temperature (20 and 60 °C), plant-solvent ratio (1:20 and 1:100, *m*/*v*), number of extractions with acetone (0 and 2), and particle size (≤710 and ≤125 µm)	16	Methanol concentration and extraction temperature had significant effects on UAE yield	[81]
*Pistacia lentiscus* leaves	Total phenols, flavonoids, and tannins	Ultrasonic bath at 39 kHz frequency and 100 W of power	2^4−1^ with a central point	Single	Temperature (5 and 25 °C), time (15 and 30 min), solvent ratio (0.06 and 0.1 L/g), ethanol concentration (50 and 75%)	9	The solvent ratio is the most important factor affecting positively the UAE process	[90]

**Table 4:** Plackett-Burman design used for UAE of bioactive compounds from plant sources.

Source	Bioactive Compound	Ultrasonic Equipment	DOE	Single or Multiple Response	Factors and Levels	Number of Runs	Results	Ref.
Hawthorn seed	Flavonoids	Ultrasonic bath at 40 kHz of frequency and 100 W of power	L15 (+1, 0, −1)	Simple	Ultrasound temperature (55 and 75 °C), time (30 ad 50 min), ethanol concentration (55 and 85%), solid-liquid ratio (1:14 and 1:22), extraction temperature (82 and 98 °C), and extraction time (1 and 2 h)	15	Ultrasonic time, ethanol concentration, and temperature were the most significant variables that influenced the UAE process	[107]
*Lonicera caerulea*	Anthocyanins	Ultrasonic bath at 40 kHz of frequency and 100 W of power	L15 (+1, −1)	Single	Solvent-liquid ratio (5:1 and 25:1), ethanol concentration (70 and 100%), formic acid concentration (0 and 1%), ultrasound bath temperature (25 and 45 °C), extraction time (10 and 30 min)	15	Liquid-solid ratio, solvent concentration, and extraction time were the most significant factors that affects the yield recovering od anthocyanins	[91]
Grape pomace	Phenolic compounds	Ultrasonic bath at 28 kHz of frequency and 600 W of power	L11 (+1, −1)	Single	Ethanol concentration (0, 40, and 80%), solid-to-liquid ratio (1:10, 1:35, and 1:60 g/mL)	11	Solvent concentration significantly influenced the extraction yield	[108]
*Ceratonia siliqua*	Polyphenols	Ultrasonic bath operating in continuous mode	L11 (+1, −1)	Multiple	Extraction time (5 and 60 °C), temperature (15 and 50 °C), solid: solvent ratio (0.05 and 0.2 g/mL), solvent concentration (0 and 100%), sonication frequency (37 and 80 kHz), sonication power (30 and 100 W), particle size (0.3 and 2 mm)	11	Extraction time and temperature were the most important factors that influenced the recovering yield of polyphenols	[73]
*Rubia sylvatica* Nakai fruit	Total anthocyanins ad total phenolics	Ultrasonic bath at 40 kHz of frequency and 600 W of power at 30 °C for 20 min	L12 (+1, −1)	Single	Ethanol concentration (30 and 40%), liquid: solid ratio (20 and 30 mg/L), ultrasound power (400 and 500 W), pH value (2 and 3), extraction temperature (50 and 60 °C), extraction time (20 and 30 min)	12	Recovering yield of bioactive compounds is dependent on experimental conditions and type of compound	[109]
*Kaempferia parviflora* Rhizomes	Methoxyflavone	Ultrasonic bath at 40 kHz of frequency and 160 W of power	L12 (+1, −1)	Single	Type of solvent (methanol and ethanol), organic solvent concentration (50 and 95%), extraction time (5 and 30 min), temperature (30 and 80 °C), solvent-to-solid ratio (10 ad 50 mL/g)	12	The most critical variables were ethanol concentration, solvent-to-solid ratio, and extraction time	[71]

**Table 5:** Box-Behnken design used for UAE of bioactive compounds from plant sources.

Source	Bioactive Compound	Ultrasonic Equipment	DOE	Single or Multiple Response	Factors and Levels	Number of Runs	Results	Ref.
*Piper betle* leaves	Total phenols and flavonoids	Ultrasonic bath at 37 kHz and 400 W of power	3^3^	Multiple	Temperature (50, 60, and 70 °C), ethanol concentration (70, 80, and 90% *v*/*v*), and solid-to-liquid-ratio (1:10, 1:20, and 1:30 g/mL)	17	Solid-to-liquid ratio had significant effects on yield	[92]
Myrtle (*Myrtus communis* L.)	Phenolic compounds and total anthocyanins	Ultrasonic probe	3^6^	Single	Solvent concentration (50–100% for phenolic and 25–75% for anthocyanins), temperature (10–60 °C), amplitude (30–70%), cycle (0.2–0.7 s), pH (2–7), and liquid-to-solid ratio (10:0.5–20:0.5 mL/g)	54	Interaction between solvent and temperature and interaction between cycle and liquid-to-solid ratio had significant effects on phenolics yield, where solvent and pH had significant effects on anthocyanins yield	[111]
Malagueta peppers	Phenolic compounds and flavonoids	NI	3^3^	Multiple	Solvent volume (8, 12, and 16 mL), time (15, 30, and 45 min), temperature (40, 50, and 60 °C)	15	The best UAE conditions were 16 mL of solvent during 15 min at 55 °C	[98]
Common centaury (*Centaurium erythraea Rafn*)	Total phenolic compounds	Ultrasonic bath at 40 kHz and 150 W of power	3^4^ with three center point	Single	Time (20, 25, and 30 min), solvent concentration (30, 50, and 70% *v*/*v*), liquid-to-solid ratio (5, 10, and 15 mL/g), temperature (40, 55, and 70 °C)	29	All extraction factors and their interaction significantly influenced the UAE yield	[4]
Yellow and Red Tamarillo fruits (*Solanum betacum*)	Phenolic compounds and flavonoids	Ultrasonic probe at 6 mm diameter and 500 W of power, amplitude of 0–100%, and pulse cycle of 2 s	3^3^ with five central point	Multiple	Time (5, 10, and 15 min), amplitude (20, 40, and 60%), solvent concentration (50, 60, and 65%)	17	All extraction factors and their interaction significantly influenced the UAE yield	[112]
Muicle (*Justicia spicigera*) leaves	Phenolic compounds	Ultrasonic probe at 7 mm diameter, 400 W and 24 kHz	3^3^	Single	Pulse cycle (0.4, 0.7, and 1 s), amplitude (40, 70 and 100%), time (2, 7, and 12 min)	15	Pulse cycle was the most important factor followed by amplitude for UAE process	[113]
*Annona muricata* by-products	Phenolic compounds	Ultrasonic probe at 7 mm diameter, 400 W and 24 kHz	3^3^	Single	Pulse cycle (0.4, 0.7, and 1 s), amplitude (40, 70 and 100%), time (5, 10, and 15 min)	15	The yield recovering depended on the composition of matrix	[114]
*Psidium cattleianum* leaves	Phenolic compounds	Ultrasonic probe at 7 mm diameter, 400 W and 24 kHz	3^3^	Single	Pulse cycle (0.4, 0.7, and 1 s), amplitude (60, 80 and 100%), time (2, 4, and 6 min)	15	Extraction time had significant effects on yield	[31]
Pomegranate peel	Total phenolics and flavonoids	Ultrasonic probe at 6 mm diameter, 500 W and 40 kHz	3^4^	Single	Pulse cycle (0.2, 0.5, and 0.8 s), amplitude (50, 65, and 80%), time (5, 10, and 15 min), methanol concentration (30, 50, and 70%)	29	Methanol concentration and amplitude has significant effect on UAE process	[115]
jabuticaba (*Myrciaria cauliflora*) fruit	Phenolic compounds and anthocyanins	Ultrasonic probe at 7 mm diameter, 200 W and 24 kHz	3^6^	Single	Methanol concentration (25, 50, and 75%), temperature (10, 40, and 70 °C), amplitude (30, 50, and 70%), cycle (2, 4.5, and 7 s), solvent-to-sample ratio (10:1.5, 15:1.5, and 20:1.5)	54	Solvent composition was the most important factor that influenced the UAE yield recovering	[57]

**Table 6:** Central composite design used for UAE of bioactive compounds from plant sources.

Source	Bioactive Compound	Ultrasonic Equipment	DOE	Single or Multiple Response	Factors and Levels	Number of Runs	Results	Ref.
Spinach roots	TPC and TFC	Ultrasonic probe of 9 mm diameter at 200 W, 20 kHz of frequency	CCD with 3 independent variables at 5 levels (−α, −1, 0, +1, + α)	Multiple	Amplitude (10, 25, 40, 55, and 70%), temperature (0, 10, 20, 30, and 40 °C), time (2, 3, 4, 5, and 5 min), ethanol concentration (0, 20, 40, 60, and 80%)	30	TPC and flavonoids yield were influenced by independent variables during extraction process	[2]
Acerola residues	Carotenoids, phenolics, and flavonoids	Ultrasonic bath at 50 kHz and 250 W of power	CCRD with 8 factorial, 6 axial, and 3 central points	Multiple	Ethanol concentration (0–99.5%), ethanol: residue ratio (1–10 mL/g), and extraction time (10–60 min)	17	All factors significantly influenced the UAE yield recovering in a bioactive compound-response manner	[116]
Safflower seed	NI	NI	CCD with 3 independent variables at 5 levels (−α, −1, 0, +1, + α)	Single	Extraction time (5–55 min), temperature (26–94 °C), and ethanol concentration (0–100%)	17	The highest extraction yield was observed applying 80% ethanol concentration for 45 min at 40 °C	[80]
*Ficaria kochii*	TPC and TFC	Ultrasonic bath at 50–60 kHz	CCRD with 3 independent variables at 5 levels (−α, −1, 0, +1, + α)	Single	Time (30–60 min), solvent-to-solid ratio (1–13%), and temperature (30–70 °C)	20	All factors significantly influenced the UAE yield recovering in a bioactive compound-response manner	[51]
Chestnut shells	Polyphenols	Ultrasound probe at 13 mm diameter and 50% of amplitude	CCD with two independent variables at 5 levels	Multiple	Time (4, 10, 25, 40, and 46 min) and temperature (34, 40, 55, 70, and 76 °C)	13	The extraction time has significant effect on UAE yield recovering, while temperature did not show significant effect	[117]
Garlic leaves	TPC and TFC	Ultrasound probe at 16 mm diameter at 20 kHz and 700 W of power	CCRD with 3 independent variables at 5 levels (−α, −1, 0, +1, + α)	Single	Ultrasound amplitude (19, 30, 45, 60, and 70%), time (1.6, 5, 10, 15, and 18.4 min), and ethanol concentration (33, 40, 50, 60, and 66.8%)	20	The highest extraction yield was observed under 50% ethanol concentration for 13 min and 53% amplitude	[20]
*Garcinia indica*	TPC and TFC	NI	CCFC with 5 independent variables at 5 levels (−α, −1, 0, +1, + α)	Single	Ultrasound intensity (46, 60, 70, 80, and 93 Wcm^2^), methanol concentration (49, 60, 67, 75, and 85%), pulse cycle (0.05, 0.2, 0.4, 0.6, and 0.88 s), particle size (0.1, 0.25, 0.625, 1, and 1.52 mm), temperature (9.3, 30, 45, 60, 80.6 °C)	48	The extraction yield was dependent on experimental conditions for both bioactive compounds	[118]
Black locust flowers	TPC	Ultrasonic bath at 40 kHz	CCD with 3 independent variables at 5 levels (−α, −1, 0, +1, + α)	Multiple	Ethanol concentration (33–67%), temperature (33–67 °C), and time (17–33 min)	17	The highest extraction yield was observed under 60% ethanol concentration for 30 min	[84]
*Sideritis raeseri*	TPC	Ultrasonic bath	CCD with 4 independent variables at five levels (−α, −1, 0, +1, + α)	Multiple	Extraction time (5, 20, 35, 50, and 65 min), ethanol concentration (10, 30, 50, 70, and 90%), solid-to-liquid ration (1:10, 1:20, 1:30, 1:40, and 1:50 g/mL), temperature (20, 35, 50, 65, and 80 °C)	30	The highest extraction yield was observed under 65% ethanol concentration for 50 min at 63 °C using a solid-to-liquid ratio of 1:40	[119]
*Triticum aestivum* seeds	TPC	Ultrasonic bath at 40 kHz and 150 W of power	CCD with 3 independent variables at 5 levels (−α, −1, 0, +1, + α)	Multiple	Ethanol concentration (33, 40, 50, 60, and 67% *v*/*v*), temperature (33, 40, 50, 60, and 67 °C), and time (17, 20, 25, 30, and 33 min)	18	The highest extraction yield was observed under 56% ethanol concentration for 28 min at 59 °C	[120]
*Ceratonia siliqua*	Polyphenols	Ultrasonic bath at 40 kHz of frequency and 160 W of power	Non-standard central composite design with α = 1.6818 for rotatability	Multiple	Solvent-to-solid ratio (0.05, 0.08, 0.2, 0.21 mL/g), ethanol concentration (0, 20, 5, 80, 100), particle size (0.3, 0.5, 1.0, and 2.0 mm)	17	The effect depended in the type of extracted polyphenol compound	[73]

NI: No information; TPC: Total phenolic content; TFC: Total flavonoid content; CCD: Central composite design; CCRD: Central composite rotatable design; CCFC: Central composite face-centered design.

**Table 7:** Taguchi design used for UAE of bioactive compounds from plant sources.

Source	Bioactive Compound	Ultrasonic Equipment	DOE	Single or Multiple Response	Factors and Levels	Number of Runs	Results	Ref.
Coffee leaves	Polyphenols	NI	L8 (2^6^)	Multiple	Ethanol concentration (0 and 60%), temperature (30 and 80 °C), ultrasound power (0 and 210 W), time (10 and 40 min), coffee leaf age (young and mature), liquid: solid ratio (10:1 and 40:1)	8	Liquid: solid ratio, ethanol concentration, and extraction temperature were the most significant factor that influenced the recovery yield of bioactive compounds	[127]
Red cabbage	Anthocyanins	Ultrasound probe at 10 mm of diameter	L9 (3^4^)	Single	Temperature (15, 30, and 45 °C), time (30, 60, and 90 min), power (50, 75, and 100 W), pulse mode (0.3, 0.65, and 1)	9	Time, temperature, and power ultrasound were the most important factors that contribute the yield extraction	[70]
Butterfly pea petals	Anthocyanins and total phenolic compound	Ultrasound bath at an output power of 160 W	L9 (3^3^)	Multiple	Extraction time (30, 45, and 60 min), temperature (40, 60, and 80 °C), liquid: solid ratio (5, 7.5, and 10 mL/g)	9	Liquid–solid ratio showed the highest contribution for recovering anthocyanin and total phenolic content	[24]
*Curcuma longa* rhizomes	Curcumin	Ultrasound bath	L9 (3^4^)	Single	Extraction time (20, 40, and 60 min), solvent viscosity (0.32, 0.6, and 1.2 cp), sieve number (10, 20, and 40), solvent volume (10, 20, and 30 mL)	9	Curcumin yield was influenced by the UAE conditions	[130]
*Azadirachta indica*	Phenolic compounds	Ultrasound probe at 2 cm of diameter and 13.5 cm height, frequency of 20 kHz and 120 W, pulse mode 5 s on/off	L16	Single	Particle size (0.15, 0.212, 0.425, and 0.6 mm), irradiation time (15, 30, 45, and 60 min), solid-to-liquid ratio (1:20, 1:30, 1:40, and 1:1:50), temperature (25, 35, 45, and 55 °C)	16	Particle size significant influence the yield recovering followed by temperature	[76]
*Hamelia patens*	Polyphenols	Ultrasound bath	L9 (3^3^)	Single	Solid: liquid ratio (1:8, 1:12, and 1:16), extraction time (10, 20, and 30 min), ethanol concentration (0, 35, and 70%)	9	Solid: liquid ratio was the most important factor that influenced the yield recovering of polyphenols followed by ethanol concentration	[131]
*Clitoria ternatea* petals	Anthocyanins	Ultrasound bath	L27 (3^3^) and S/N ratio	Single	Time (30, 40, and 50 min), temperature (40, 50, and 60 °C), solvent-to-liquid ratio (10:1, 20:1, and 30:1 mL/g)	27	The optimum conditions for UAE of anthocyanins were 50 °C at 10:1 mL/g for 30 min	[132]

NI: No information; S/N ratio: Signal-to-Noise ratio.

**Table 8:** Mixture designs used for extracting bioactive compounds from plant sources.

Source	Bioactive Compound	Ultrasonic Equipment	DOE	Single or Multiple Response	Factors and Levels	Number of Runs	Results	Ref.
*Physalis angulata*	Polyphenols	Ultrasound bath at 50/60 Hz and 90 W of power for 10 min at 30 °C	SCD	Single	Water (0–100%), methanol (0–100%), ethanol (0–100%), sonication time (15 min), extractor volume (15 mL)	7	The best proportions of solvents were 57% water, 35% ethanol, and 8% methanol	[136]
Cashew apple	Carotenoids	Ultrasound bath at 40 kHz and 80 W of power	SCD	Single	Acetone (0–100%), ethanol (0–100%), petroleum ether (0–100%), methanol (0–100%)	15	The best proportions of solvents were 44% acetone and 56% methanol	[137]
*Mauritia flexuosa*	Carotenoids	Ultrasound bath at 40 kHz and 80 W of power	LSD	Multiple	Acetone, ethanol, methanol, acetonitrile	25	The best proportions of solvents were 75% acetone and ethanol 25%	[134]
Pineapple by-product	Polyphenols	NI	SCD	Single	Water (0–100%), ethanol (0–100%), and acid solution 1 mol L^−1^ HCl (0–100%)	13	The highest polyphenol yield was obtained using ethanol and acid solution in a proportion of 50:50	[138]
Moroccan *Pimpinella anisum*	Polyphenols and flavonoids	Ultrasound bath at 37 kHz and 100 W of power	SCD	Multiple	Water, ethanol, methanol, dichloromethane, chloroform, acetone, ethyl acetate, hexane, butanol and acetonitrile	12	The best proportions of solvents were 44% water, 22% ethanol, and 34% methanol	[135]
*Taraxacum assemanii*	Polyphenols	Ultrasound bath at 35 kHz	SCD	Single	Ethanol (0–100), methanol (0–100), water (0–100)	14	Ethanol-water (68:32) were the best proportion of extraction solvent	[133]
*Eugenia uniflora* leaves	Polyphenols	NI	SCD	Multiple	Water, methanol, ethanol and acetone	15	The best proportions of solvents were 46% water, 13% methanol, 18% ethanol, and 23% acetone	[139]
*Capsicum frutescens*	Polyphenols	Ultrasound bath	SCD	Multiple	Ethanol (0–100), methanol (0–100), water (0–100)	10	The best mixture proportion was 95% ethanol and water 5%	[98]
Mango peel	Polyphenols	Ultrasound probe at 2 cm diameter	SCD	Single	Ethanol, acetone, hexane	NI	The best mixture proportion was 60% ethanol and 40% acetone	[74]

SCD: Simplex-Centroid design; LSD: Lattice-Simplex design NI: No information.

**Table 9:** D-optimal design used for UAE of bioactive compounds from plant sources.

Source	Bioactive Compound	Ultrasonic Equipment	DOE	Single or Multiple Response	Factors and Levels	Number of Runs	Results	Ref.
Walnut male flowers	Phenolic compounds and flavonoids	Ultrasound bath	Three factors, three levels D-optimal design	Multiple	Extraction time (10, 30, and 50 min), solvent type (methanol, ethanol, and acetone), and water in solvent (20, 40, and 60% *v*/*v*)	21	The higher extraction yield was performed after 30 min of extraction containing 40% water in acetone	[86]
*Cynara sco-* *Lymus* leaves	Phenolics and flavonoids	Ultrasound probe at 13 mm of diameter, and 500 W of power at 20 kHz of frequency	Two factors D-optimal design	Multiple	Extraction time (20–60 min), Ultrasound amplitude (30–80%)	19	The best extraction conditions were 20.05 min of extraction time and 65.02% of ultrasonic amplitude	[145]
*Echinacea purpurea using*	Polyphenols	Ultrasound bath at 320 W of power and 35 kHz of frequency for 30 min	Four factor D-optimal design	Multiple	Temperature (25–75 °C), sonication time (0–60 min), solvent concentration (0–100%), and solvent type (methanol and ethanol)	34	The optimal UAE conditions were 41.70% methanol at 75 °C for 51.8 min	[144]
Grapefruit leaves	Phenolic compounds	Ultrasound probe at 20 kHz of frequency and 125 W of power	Six factors D-optimal design	Multiple	Ethanol concentration (0–50%), extraction time (15–60 min), temperature (25–50 °C), solid: liquid ratio (50–100 g/L), ultrasound power density (0.25–0.5 kW/L), probe type (thin and thick)	34	The optimal UAE conditions were ethanol concentration of 10.80% at 30.37 °C for 58.52 min	[141]
Wild thyme aerial parts	Phenolic compounds	Ultrasound probe at 6 mm of diameter	Three factor D-optimal design	Single	Time (1, 3, 5, 7, and 10 min), ultrasound amplitude (20, 30, and 40%), ethanol concentration (30, 50, and 70%)	19	The optimal UAE conditions were time 5 min, amplitude 30%, and ethanol concentration 50%	[143]
Olive leaves	Oleuropin	Ultrasound probe at 13 mm of diameter and 20 kHz of frequency	Five factors D-optimal design	Multiple	Amplitude (32–89%), sonication time (1–15 min), ethanol or methanol concentration (50–80%), probe position (1.5–4 cm), duty cycle (0.3–1%), solvent: solid ratio 12.80 mg/L, temperature 30 °C	31	The optimum UAE conditions were amplitude 81.91%, time 14.22 min, MeOH 76.97%, probe position 3.89 cm, duty-cycle 0.93%	[142]

**Table 10:** Combined designs investigated for extracting bioactive compounds from plant sources.

Source	Bioactive Compound	Screening DOE	Optimizing DOE	RSM	Mathematical Model	Ref.
Ripe carob pods	Polyphenols	Placket-Burman	Non-standard central composite	RSM	Quadratic model	[73]
*Pistacia lentiscus* Leaves	Polyphenols	Fractional factorial design	Box-Behnken	RSM	Quadratic model	[90]
*Cecropia* sp.	Polyphenols	Fractional factorial	Central composite	RSM	Quadratic model	[81]
Sour cherries	Polyphenols and anthocyanins	Fractional factorial	Face-centered central composite	RSM	Quadratic model	[106]
Haskap berries	Anthocyanins	Placket-Burman	Box-Behnken	RSM	Linear and quadratic models	[91]
Hawthron seed	Flavonoids	Placket-Burman	Box-Behnken	RSM	Linear and quadratic models	[107]
Grape pomace	Phenolic compounds	Placket-Burman	Face-centered central composite	RSM	Quadratic model	[108]
*Kaempferia parviflora* Rhizomes	Methoxyflavon	Placket-Burman	Box-Behnken	RSM	Linear and quadratic models	[71]
*Rubia sylvatica*	Anthocyanins	Placket-Burman	Box-Behnken	RSM	Linear and quadratic models	[109]
Coffee leaves	Phenolic compounds	Taguchi design	Box-Behnken	RSM	Quadratic model	[127]
*Mauritia flexuosa*	Carotenoids	Simplex-lattice	Central composite	RSM	Linear, quadratic, and cubic models	[134]
Malagueta peppers	Phenolic compounds	Full factorial	Box-Behnken	RSM	Quadratic model	[98]
*Croton heliotropiifolius* Kunth leaves	Phenolic compounds	Full factorial	Doehlert	RSM	Quadratic model	[147]

DOE: Design of experiments; RSM: Response surface methodology.

**Table 11:** Advantages and limitations of DOEs for UAE of bioactive compounds from plant sources.

Design of Experiment	Advantages	Limitations
Full factorial	Robust DOE It is possible to evaluate the main and the interaction effects clearly	Number of factors should be 2 to 5. Substantial increase in the number of experiments as the number of factors increases. Complexity in interpreting complex interactions. Confusion issues may arise when there are interactions. Difficulty handling categorical factors.
Fractional factorial	It is recommended when the number of factors exceeds 4 Allows for study of interactions and quadratic effects within variables Reduced number of experimental runs compared to full factorial design	Designs with high degree of aliasing may result in high collinearity between variables. May lose important information by omitting some combinations. Not suitable for all experiments due to the design fraction. Difficulty in studying higher-order interactions. Choosing the appropriate fraction can be challenging.
Plackett-Burman	It is a useful tool for initiating the optimization process by screening a substantial number of factors (>4) Eliminate non-significant variables from the models	The aliasing pattern is highly complex, each main effect is aliased with every two-way interaction not involving that effect. Lack of fit is difficult to assess, and first-order effects may be confounded with interaction effects. Limited in its ability to study non-linear responses. Does not provide information on the influence of categorical factors.
Box-Behnken	Allows for study of interactions and quadratic effects within variables Reduced number of experimental runs compared to full factorial design	Substantial increase in the number of experiments as the number of factors increases. At least 3 factors and 3 levels are required. It does not examine borderline regions of experiment factors. Cannot handle categorical factors. The choice of central points can affect the accuracy of estimates.
Central composite	It no need for a three-level factorial design for building a second-order quadratic model Allows for study of interactions and quadratic effects within variables It contains the extreme factor combinations Maximum information in a minimum experimental trial	The star points are outside the hypercube. Depending upon the Design, the squared terms in the model will not be orthogonal to each other. Inability to estimate individual interaction terms Efficiency may decrease in the presence of interactions. Sensitive to the choice of axial and central points.
Taguchi	Robust DOE It is a screening tool for identifying the significant factors that affect the process A good amount of data can be obtained with lesser resources	It exhibited difficulty in accounting for interactions between parameters. It is not appropriate in dynamically changing processes. Limited in terms of flexibility for some types of responses. Design robustness may depend on the appropriate choice of factor levels.
Simplex-Centroid	It is widely used for obtaining formulations It minimizes the model error and the number of required experiments	Not suitable for experiments with many factors. Efficiency may decrease if factors are highly correlated. Does not allow the evaluation of complex interactions. Interpretation of effects can be complicated.
D-optimal	Significant reduction in number of experimental runs Allow the study of multiple combinations of multilevel factors, independently if the number of variable levels of factors is different in the same experimental design	May require use of extensive computational resources Requires prior knowledge of effect variances. Does not guarantee a unique design, which can lead to suboptimal solutions. Interpretation can be challenging for experimenters unfamiliar with optimal design theory. Implementation can be costly and require additional resources.
Doehlert	It is enables to examinate multiple variables with different levels within a single matrix, reducing the number of experiments	It does not have any of the properties of the response surface matrices that include isovariance by rotation, orthogonality, and uniform precision. Not efficient when the number of factors is large. Limited in terms of handling categorical factors. Interpretation can be complicated for complex responses.

Source: [58,149,150,151,152,153].

## Data Availability

The data presented in this review are available on request from the corresponding authors.
